# Preliminary Report on the Use of Anesthol, a New General Anesthetic Based on Schleich’s Principles, as Introduced by Dr. Willy Meyer of New York*Read before the West Texas Medical Association June 25, 1903.

**Published:** 1903-12

**Authors:** W. B. Russ, T. P. Lloyd

**Affiliations:** San Antonio, Texas; San Antonio, Texas


### TEXAS MEDICAL JOURNAL.

ESTABLISHED JULY, 1885.

PUBLISHED MONTHLY.—SUBSCRIPTION $1.00 A YEAR.

VOL. XIX. AUSTIN, DECEMBER, 1903. No. 6.

### Original Contributions.

For Texas Medical Journal.

### Preliminary Report on the Use of Anesthol, a New General Anesthetic Based on Schleich's Principles, as Introduced by Dr. Willy Meyer of New York.*

BY W. B. RUSS, M. D., AND T. P. LLOYD, M. D., SAN ANTONIO, TEXAS.

Commencing with the theory that in the case of general anesthetics the amount of narcotic substance evaporating within a given time depends upon (1), the temperature within the body, and (2), the boiling point of the narcotic, Schleich reasoned that, in order to be readily eliminated, the boiling point of an anesthetic must closely approximate body temperature. This he illustrated by the results observed during the administration of chloroform and sulphuric ether. The boiling point, or "maximum of evaporation," of chloroform being 149° F., more of this poison is taken in during inspiration, than is eliminated during expiration, with the result that the excess remains to overtax the other paranchymatous organs. Sulphuric ether, on the other hand, having a boiling point of only 93.2° F., when introduced into the lungs (where the temperature is 100.4°), expands and often seriously cripples the alveoli by overdistension of their walls. This condition prevents the ready elimination of carbon dioxide and produces a primary cyanosis and certain distressing reflex symptoms with which we are all familiar. Though, after a time, the accumulation of carbon dioxide within the blood overcomes the abnormal inter-alveolar tension, there is

*Read before the West Texas Medical Association June 25, 1903.

never, at any time during the administration of this anesthetic, a proper diffusion of gases within the lungs. Thus it is only with the assistance of the tension produced in the parenchyma of the lungs in this manner, that a part of the ether is forced into the blood, and, ether narcosis made possible. In other words, in the case of chloroform administration a far greater amount than is needed enters the circulation, and the surplus is eliminated slowly and with great difficulty, the heart, kidneys, liver, and other organs being often seriously damaged. Ether, on the other hand, can only enter the circulation to produce anesthesia by aid of a carbonic acid poisoning, the result of abnormal tension produced within the lungs by expansion of the ether vapor within their alveoli. With these facts in mind, Schleich sought to devise a combination that would have a boiling point closely approximating body temperature, which would make possible a ready diffusion of gases within the lungs, and insure a rapid elimination of the anesthetic. After some experimentation he announced the now famous "Schleich Mixtures," consisting of chloroform, sulphuric ether, and petrolic ether (or benzine). This combination, though highly praised by some, did not, on the whole, give satisfaction and an explanation of the untoward results sometimes following its use was readily forthcoming when, in 1898, at the request of Dr. Meyer, Dr. S. J. Meltzer of New York investigated petrolic ether and demonstrated that it is not only inert as to anesthetic properties, but is a highly poisonous substance when administrated pure to animals, producing, in large doses, death, with symptoms of tetanus.

Having great faith in the correctness of Schleich's reasoning, Dr. Meyer, some four years ago, undertook to find a non-toxic drug of low specific gravity and possessed of anesthetic properties with which to replace the petrolic ether in Schleich's mixture. Finally, at the suggestion of Dr. H. P. Weidig of Newark, N. J., he added ethyl chlorid, the boiling point of which is 59° F. (15° C.), to the chloroform-ether mixture, in the proportion of 17 per cent of ethyl chlorid to 83 per cent chloroform-ether mixture, thus giving the combination a boiling point of 104° F. (40° C.). Dr. Weidig, in 1898, it will be remembered, demonstrated that chloroform and ether, when combined in the proportion of their molecular weights, unite to form an entirely new substance, and not a simple mechanical mixture, as is commonly supposed.

For the past four years Dr. Meyer has used the above combination, which he calls "Anesthol," in his practice, with the greatest possible success, and he claims for it that it combines all of the good effects of chloroform and ether, and possesses decided advantages over both.

Being much impressed with Dr. Meyer's report, it was determined, some weeks ago, to give the new anesthetic a trial. However, owing to the late arrival of our supply, we have had an opportunity of using it in only eight cases, of which the following are typical:

Case 1.—Boy 15 years old, phimosis, pulse 80, respiration 21. At the beginning of the first stage the patient's extreme nervousness ran the pulse up to 120, but within three or four minutes, it became normal in every respect, and so continued throughout the operation. There was no excitement during the second stage; no salivation or bronchorrœa. The respiration remained perfectly normal throughout the entire time. The pupils were slightly contracted. Complete muscular relaxation was secured within ten minutes and was maintained until the completion of the operation. Within five minutes after the anesthetic had been discontinued the patient vomited once, and then suddenly regained consciousness in full possession of his intellectual faculties. He immediately asked to be allowed to get out of bed, and claimed to be free from nausea, dizziness, headache, and muscular weakness. About twenty grams of the anesthetic were used and the patient was under its influence 26 minutes.

Case 2.—Female, 30 years old, endometritis, pulse 80, respiration 18. Time required to secure complete surgical anesthesia, 10 minutes; time under the influence of the anesthetic, forty minutes; amount of anesthol used, 30 grams. The history of this case during the administration of the anesthetic was about the same as the preceding one, except that the patient vomited twice shortly after regaining consciousness.

The other cases present similar histories. In every instance the anesthetic seemed to exert but little, if any, influence upon the pulse and respiration, and at no time has a patient's condition been such as to excite any uneasiness whatever. In every instance, after the return of consciousness, the patient has seemed to be as bright as if awakened from a refreshing sleep. Vomiting has not been persistent in any except one instance. In this case, however, a preliminary hypodermic injection of morphia had been given, as advised by Dr. Meyer. The patient complained of persistent nausea and some headache for several hours after the operation.

Our limited experience with anesthol has been attended with such happy results that we feel justified in recommending it for general use, and in attributing to it the following advantages:

1. It seems to have but little, if any, depressing effect upon the circulatory and respiratory systems.

2. Its administration being attended with practically no anesthetic shock; patients return to consciousness in full possession of their intellectual powers and free from headache, dizziness, muscular weakness, etc.

3. Its administration is not disagreeable to the patient.

4. The introduction of anesthesia is gradual and progressive. No sudden and alarming changes in the condition of the pulse and respiration occur.

5. There are practically no contra-indications to its use.

We are indebted to Dr. G. Graham Watts for valuable assistance with three of the cases, and for furnishing one subject.

